# Development and validation of a new diabetes index for the risk classification of present and new-onset diabetes: multicohort study

**DOI:** 10.1038/s41598-021-95341-8

**Published:** 2021-08-03

**Authors:** Shinje Moon, Ji-Yong Jang, Yumin Kim, Chang-Myung Oh

**Affiliations:** 1 Department of Endocrinology and Metabolism, Hallym University College of Medicine, Chuncheon, Republic of Korea; 2 Division of Cardiology, National Health Insurance Service Ilsan Hospital, Goyang, Republic of Korea; 3 Department of Biomedical Science and Engineering, Gwangju Institute of Science and Technology, Gwangju, Republic of Korea

**Keywords:** Endocrinology, Machine learning

## Abstract

In this study, we aimed to propose a novel diabetes index for the risk classification based on machine learning techniques with a high accuracy for diabetes mellitus. Upon analyzing their demographic and biochemical data, we classified the 2013–16 Korea National Health and Nutrition Examination Survey (KNHANES), the 2017–18 KNHANES, and the Korean Genome and Epidemiology Study (KoGES), as the derivation, internal validation, and external validation sets, respectively. We constructed a new diabetes index using logistic regression (LR) and calculated the probability of diabetes in the validation sets. We used the area under the receiver operating characteristic curve (AUROC) and Cox regression analysis to measure the performance of the internal and external validation sets, respectively. We constructed a gender-specific diabetes prediction model, having a resultant AUROC of 0.93 and 0.94 for men and women, respectively. Based on this probability, we classified participants into five groups and analyzed cumulative incidence from the KoGES dataset. Group 5 demonstrated significantly worse outcomes than those in other groups. Our novel model for predicting diabetes, based on two large-scale population-based cohort studies, showed high sensitivity and selectivity. Therefore, our diabetes index can be used to classify individuals at high risk of diabetes.

## Introduction

Diabetes mellitus is a chronic metabolic disorder characterized by disrupted glucose homeostasis, resulting from increased insulin resistance and/or impaired insulin secretion. People with diabetes mellitus are predisposed to metabolic disorders, such as cardiovascular disease (CVD), which affects 32.2% of all people with diabetes mellitus globally. Moreover, their complications are leading causes of morbidity and mortality^1,2^. The prevalence and socioeconomic burden of diabetes are rapidly increasing worldwide. Approximately 1 in 11 adults have diabetes, and 90% of people with diabetes have type 2 diabetes mellitus^2^.

Previous large-scale studies suggest that diet and lifestyle modifications can prevent or delay the development of diabetes mellitus in high-risk individuals by Refs.^2,3^. The Diabetes Prevention Program conducted in the Unites States reported that lifestyle modification reduced the incidence of diabetes mellitus by 58% compared with control after a 2.8-year mean follow-up^4^. Toshikazu et al. also demonstrated that lifestyle modification reduced the overall relative risk of diabetes mellitus by 44.1% in Japan^5^. Clinical studies conducted in China^6^ and India^7^ have reported 42% and 38% risk reductions, respectively.

Therefore, developing risk prediction models for diabetes mellitus and identifying high-risk individuals have become a challenging issue in clinical research. To explore the risk factors and formulate predictive models for diabetes development, machine learning techniques have been widely used^8^. These methods help researchers discover unknown significant figures and solve scientific problems from large quantities of datasets^9,10^. In the fields of medical science and healthcare, machine learning provides useful classification and prediction models with high accuracy^11^. Recently, Hang Lai et al. proposed a risk prediction model with 84.7% area under the receiver operating characteristic curve (AUROC) from 13,309 Canadian patients^12^. Furthermore, Maniruzzaman et al. built a classifying model that yielded 94.25% accuracy for the prediction of diabetes mellitus from an American diabetes dataset^13^.

In this study, we aimed to propose a novel diabetes index based on machine learning techniques for diabetes mellitus with high accuracy from two large community-based cohort studies. We formulated a risk classification model using logistic regression to measure the probability of diabetes presence, based on non-diabetic participants’ demographic information and laboratory data from the Korea National Health and Nutrition Examination Survey (KNHANES). Thereafter, we externally validated this model by predicting new-onset diabetes mellitus in a large prospective cohort study known as the Korean Genome and Epidemiology Study (KoGES).

## Results

### Baseline characteristics from the KNHANES

Table 1 show the general characteristics from the KNHANES. These depict the derivation and internal validation datasets, respectively, according to gender and diabetes. Subjects with diabetes were older than those without in both datasets. In the derivation dataset, diabetes prevalence was 4.9% in men and 3.8% in women. The prevalence of obesity (Body mass index, BMI ≥ 25 kg/m^2^) was 38% in men (38% in normal and 38% in diabetes) and 28.1% in women (27.3% in normal and 47.3% in diabetes). In the internal validation dataset, diabetes prevalence was 4.6% in men and 3.9% in women. The prevalence of obesity (BMI ≥ 25 kg/m^2^) was 40.8% in men (40.6% in normal and 44.5% in diabetes) and 27.6% in women (26.8% in normal and 46% in diabetes). Subjects with diabetes in both datasets exhibited lower socioeconomic status and education, higher fasting glucose levels, as well as higher incidence of glycosuria, hypertension, and dyslipidemia than did subjects without diabetes.

**Table 1:** General characteristics of training set (2013–16 KNHANES) and testing set (2017–2018) according to gender and diabetes.

	Men			Women		
	Normal	Diabetes	P-value	Normal	Diabetes	P-value
**2013–16 KNHANES**						
N	5751	300		8590	336	
Age, y	49.3 ± 0.21	65.2 ± 0.59	< 0.001	48.6 ± 0.17	66.4 ± 0.57	< 0.001
**Income, n**			0.019			0.342
Quartile 1	1338 (23.3)	77 (25.7)		1980 (23.1)	89 (26.5)	
Quartile 2	1446 (25.1)	95 (31.7)		2181 (25.4)	88 (26.2)	
Quartile 3	1465 (25.5)	63 (21)		2211 (25.7)	84 (25)	
Quartile 4	1502 (26.1)	65 (21.7)		2218 (25.8)	75 (22.3)	
**Education, n**			< 0.001			< 0.001
Elementary	804 (14)	101 (33.7)		1921 (22.4)	201 (59.8)	
Middle school	586 (10.2)	54 (18)		826 (9.6)	58 (17.3)	
High school	2033 (35.4)	81 (27)		2844 (33.1)	57 (17)	
College	2328 (40.5)	64 (21.3)		2999 (34.9)	20 (6)	
Smoking, pack years	13.6 ± 0.23	22.3 ± 1.25	< 0.001	0.6 ± 0.04	1.2 ± 0.3	0.042
Alcohol, g/week	119.5 ± 2.28	79.5 ± 8.06	< 0.001	28.4 ± 0.86	6 ± 1.18	< 0.001
Sleep duration, h	6.9 ± 0.02	7.1 ± 0.09	0.001	6.9 ± 0.01	6.4 ± 0.09	< 0.001
Hypertension, n	1012 (17.6)	174 (58)	< 0.001	1364 (15.9)	204 (60.7)	< 0.001
Dyslipidemia, n	321 (5.6)	87 (29)	< 0.001	723 (8.4)	128 (38.1)	< 0.001
Cardiovascular disease, n	193 (3.4)	55 (18.3)	< 0.001	188 (2.2)	37 (11)	< 0.001
Systolic BP, mmHg	120.2 ± 0.2	123.3 ± 0.98	0.002	115 ± 0.19	126.1 ± 0.99	< 0.001
Diastolic BP, mmHg	77.8 ± 0.14	71.8 ± 0.57	< 0.001	73.3 ± 0.1	71.4 ± 0.51	< 0.001
**Body mass index, n**			0.999			< 0.001
Normal (< 23 kg/m^2^)	2044 (35.5)	107 (35.7)		4421 (51.5)	95 (28.3)	
Pre-obesity (23 ≤ and < 25 kg/m^2^)	1519 (26.4)	79 (26.3)		1824 (21.2)	82 (24.4)	
Obesity (≥ 25 kg/m^2^)	2188 (38)	114 (38)		2345 (27.3)	159 (47.3)	
Waist circumference, cm	85.1 ± 0.12	88.2 ± 0.51	< 0.001	78.1 ± 0.1	85.3 ± 0.48	< 0.001
Fasting glucose, mg/dL	96.2 ± 0.13	107.2 ± 0.81	< 0.001	93.2 ± 0.1	106.3 ± 0.74	< 0.001
Glycosuria, n	33 (0.6)	29 (9.7)	< 0.001	20 (0.2)	24 (7.1)	< 0.001
Total cholesterol, mg/dL	189.8 ± 0.46	164.8 ± 2.03	< 0.001	192.2 ± 0.38	177.3 ± 2.35	< 0.001
Triglyceride, mg/dL	155.7 ± 1.66	147.9 ± 5.97	0.21	112.6 ± 0.87	143.6 ± 4.78	< 0.001
White blood cell, E3/μL	6.6 ± 0.02	6.9 ± 0.1	0.003	5.9 ± 0.02	6.7 ± 0.1	< 0.001
Hemoglobin, g/dL	15.3 ± 0.02	14.2 ± 0.08	< 0.001	13.1 ± 0.01	12.8 ± 0.07	< 0.001
Creatinine, mg/dL	0.999 ± 0.0038	1.199 ± 0.0654	0.006	0.699 ± 0.0023	0.801 ± 0.014	< 0.001
**2017–18 KNHANES**						
N	4015	181		5212	203	
Age, y	48.6 ± 0.26	66.9 ± 0.76	< 0.001	49.6 ± 0.22	67.8 ± 0.72	< 0.001
**Income, n**			0.412			0.02
Quartile 1	928 (23.1)	51 (28.2)		1233 (23.7)	60 (29.6)	
Quartile 2	1013 (25.2)	41 (22.7)		1320 (25.3)	58 (28.6)	
Quartile 3	1032 (25.7)	47 (26)		1289 (24.7)	50 (24.6)	
Quartile 4	1042 (26)	42 (23.2)		1370 (26.3)	35 (17.2)	
**Education, n**			< 0.001			< 0.001
Elementary	445 (11.1)	63 (34.8)		1066 (20.5)	119 (58.6)	
Middle school	362 (9)	34 (18.8)		476 (9.1)	29 (14.3)	
High school	1381 (34.4)	54 (29.8)		1594 (30.6)	39 (19.2)	
College	1827 (45.5)	30 (16.6)		2076 (39.8)	16 (7.9)	
Smoking, pack years	12.9 ± 0.26	24.1 ± 1.7	< 0.001	0.6 ± 0.06	1.3 ± 0.48	0.187
Alcohol, g/week	119.5 ± 2.63	89.4 ± 11.52	0.011	32.7 ± 1.14	12.1 ± 4.17	< 0.001
Sleep duration, h	7.2 ± 0.02	7.4 ± 0.11	0.061	7.2 ± 0.02	7.2 ± 0.11	0.944
Hypertension, n	721 (18)	120 (66.3)	< 0.001	866 (16.6)	140 (69)	< 0.001
Dyslipidemia, n	310 (7.7)	82 (45.3)	< 0.001	573 (11)	102 (50.2)	< 0.001
Cardiovascular disease, n	178 (4.4)	28 (15.5)	< 0.001	113 (2.2)	28 (13.8)	< 0.001
Systolic BP, mmHg	120 ± 0.23	124.9 ± 1.17	< 0.001	115.7 ± 0.24	127.9 ± 1.16	< 0.001
Diastolic BP, mmHg	78.2 ± 0.16	71.2 ± 0.77	< 0.001	73.8 ± 0.13	71.9 ± 0.67	0.006
**Body mass index, n**			0.081			< 0.001
Normal (< 23 kg/m^2^)	1342 (33.4)	46 (25.4)		2746 (52.7)	57 (28.1)	
Pre-obesity (23 ≤ and < 25 kg/m^2^)	1038 (25.9)	52 (28.7)		1071 (20.5)	50 (24.6)	
Obesity (≥ 25 kg/m^2^)	1635 (40.7)	83 (45.9)		1395 (26.8)	96 (47.3)	
Waist circumference, cm	85.9 ± 0.14	88.9 ± 0.6	< 0.001	77.7 ± 0.13	84.9 ± 0.6	< 0.001
Fasting glucose, mg/dL	96.8 ± 0.16	109.4 ± 0.91	< 0.001	93.6 ± 0.13	107.1 ± 0.95	< 0.001
Glycosuria, n	31 (0.8)	27 (14.9)	< 0.001	11 (0.2)	16 (7.9)	< 0.001
Total cholesterol, mg/dL	193.7 ± 0.57	155.3 ± 2.42	< 0.001	196 ± 0.51	165.4 ± 2.72	< 0.001
Triglyceride, mg/dL	154.9 ± 1.89	137.6 ± 5.9	0.006	108.9 ± 0.99	133.6 ± 6.22	< 0.001
White blood cell, E3/μL	6.5 ± 0.03	6.8 ± 0.13	0.019	5.8 ± 0.02	6.3 ± 0.12	< 0.001
Hemoglobin, g/dL	15.3 ± 0.02	14.4 ± 0.11	< 0.001	13.1 ± 0.02	12.8 ± 0.09	0.001
Creatinine, mg/dL	0.9 ± 0	1.1 ± 0.03	< 0.001	0.7 ± 0	0.7 ± 0.01	0.003

Continuous and categorical variables are described as mean ± standard error and number (percent), respectively.

P-values are measured using nominal population, not weighted population.

P-values of continuous and categorical variables are measured by Student t-test and Chi-squared test, respectively.

*KNHANES* Korea National Health and Nutrition Examination Survey, *BP* blood pressure.

### Feature selection and classification model by logistic regression

Based on literature review, we identified about 40 candidate risk factors (Supplementary Table 1), in 20 variables present in both KNHANES and KoGES. Table 2 displayed the selection process by means of a univariate LR in men and women, respectively. All 20 features from Model 1 were selected as candidate variables for univariate analysis in Model 2. By means of multivariate analysis (Models 2 and 3), we identified 16 and 18 variables as diabetes risk factors to be utilized as the input features for formulating the classification model in men and women, respectively. Thereafter, based on these variables, we generated a gender-specific diabetes classification model using LR. Note that the feature selection and the formulation of the prediction model were conducted using only the derivation dataset.

**Table 2:** Backward stepwise logistic regression of men and women in training set.

	Univariate LR	Model 1 (multivariate LR)	Model 2 (multivariate LR)
**Men**			
Age, y	1.077 (1.071–1.083)	1.033 (1.023–1.042)	1.037 (1.028–1.045)
Income	0.892 (0.835–0.952)	0.89 (0.819–0.968)	0.869 (0.803–0.939)
Education^a^	0.559 (0.524–0.596)	0.935 (0.854–1.024)	NA
Smoking, pack years	1.293 (1.244–1.345)	1.041 (0.996–1.087)	NA
Alcohol, g/week^b^	0.902 (0.881–0.923)	0.955 (0.929–0.982)	0.96 (0.934–0.987)
Sleep duration, h	1.159 (1.094–1.227)	1.092 (1.028–1.161)	1.094 (1.029–1.163)
Hypertension^c^	7.636 (6.577–8.866)	1.52 (1.246–1.853)	1.51 (1.239–1.842)
Dyslipidemia^c^	8.657 (7.301–10.266)	2.668 (2.143–3.32)	2.647 (2.127–3.294)
Cardiovascular disease^c^	9.303 (7.58–11.416)	1.583 (1.222–2.05)	1.625 (1.256–2.103)
Systolic BP, mmHg^b^	3.437 (2.279–5.184)	0.489 (0.273–0.876)	0.497 (0.278–0.89)
Diastolic BP, mmHg^b^	0.064 (0.045–0.092)	0.526 (0.309–0.894)	0.512 (0.301–0.87)
Body mass index^d^	1.089 (1–1.186)	0.771 (0.667–0.892)	0.77 (0.666–0.89)
Waist circumference, cm	1.044 (1.036–1.052)	1.051 (1.037–1.065)	1.051 (1.037–1.065)
Fasting glucose, mg/dL	1.106 (1.098–1.113)	1.09 (1.081–1.099)	1.09 (1.081–1.099)
Glycosuria^c^	20.94 (15.264–28.727)	6.557 (4.278–10.048)	6.61 (4.311–10.134)
Total cholesterol, mg/dL^b^	0.06 (0.047–0.078)	0.144 (0.106–0.196)	0.143 (0.105–0.194)
Triglyceride, mg/dL^b^	0.993 (0.913–1.079)	NA	NA
White blood cell, E3/μL^b^	1.659 (1.357–2.03)	1.834 (1.447–2.324)	1.917 (1.517–2.421)
Hemoglobin, g/dL	0.527 (0.5–0.556)	0.685 (0.637–0.736)	0.686 (0.639–0.738)
Creatinine, mg/dL	1.43 (1.299–1.575)	1.236 (1.141–1.339)	1.236 (1.141–1.338)
**Women**			
Age, y	1.089 (1.083–1.096)	1.048 (1.037–1.058)	1.053 (1.045–1.061)
Income	0.918 (0.861–0.979)	0.976 (0.902–1.056)	NA
Education^a^	0.417 (0.389–0.446)	0.924 (0.833–1.026)	NA
Smoking, pack years	1.083 (0.997–1.176)	NA	NA
Alcohol, g/week^b^	0.763 (0.733–0.793)	0.922 (0.884–0.963)	0.923 (0.884–0.963)
Sleep duration, h	0.772 (0.734–0.813)	0.892 (0.845–0.941)	0.888 (0.842–0.937)
Hypertension^c^	8.63 (7.456–9.989)	1.601 (1.316–1.947)	1.585 (1.307–1.924)
Dyslipidemia^c^	7.357 (6.314–8.573)	1.647 (1.354–2.005)	1.621 (1.333–1.972)
Cardiovascular disease^c^	5.511 (4.272–7.11)	0.912 (0.668–1.244)	NA
Systolic BP, mmHg^b^	18.489 (13.541–25.245)	1.04 (0.606–1.785)	NA
Diastolic BP, mmHg^b^	0.514 (0.358–0.739)	0.385 (0.238–0.623)	0.399 (0.263–0.605)
Body mass index^d^	1.908 (1.757–2.072)	0.881 (0.764–1.016)	NA
Waist circumference, cm	1.08 (1.073–1.088)	1.028 (1.014–1.041)	1.02 (1.011–1.03)
Fasting glucose, mg/dL	1.122 (1.115–1.13)	1.093 (1.085–1.102)	1.094 (1.085–1.102)
Glycosuria^c^	32.528 (22.296–47.456)	14.661 (9.182–23.411)	15.074 (9.475–23.979)
Total cholesterol, mg/dL^b^	0.15 (0.115–0.196)	0.108 (0.078–0.15)	0.108 (0.078–0.149)
Triglyceride, mg/dL^b^	1.856 (1.711–2.015)	1.314 (1.162–1.486)	1.306 (1.156––1.476)
White blood cell, E3/μL^b^	3.366 (2.812–4.03)	2.684 (2.159–3.338)	2.698 (2.172–3.351)
Hemoglobin, g/dL	0.809 (0.767–0.854)	0.734 (0.686–0.784)	0.73 (0.684–0.779)
Creatinine, mg/dL	1.682 (1.435–1.971)	1.063 (0.836–1.351)	NA

*LR* logistic regression.

^a^Elementary (reference: 1)/Middle school (coded as 2)/High school (coded as 3)/College (coded as 4).

^b^The variable is log-transformed.

^c^Absence of status (reference: 0)/Presence of status (coded as 1).

^d^Normal (reference: 1)/Pre-obesity (coded as 2)/Obesity (coded as 3).

**Figure 1:**
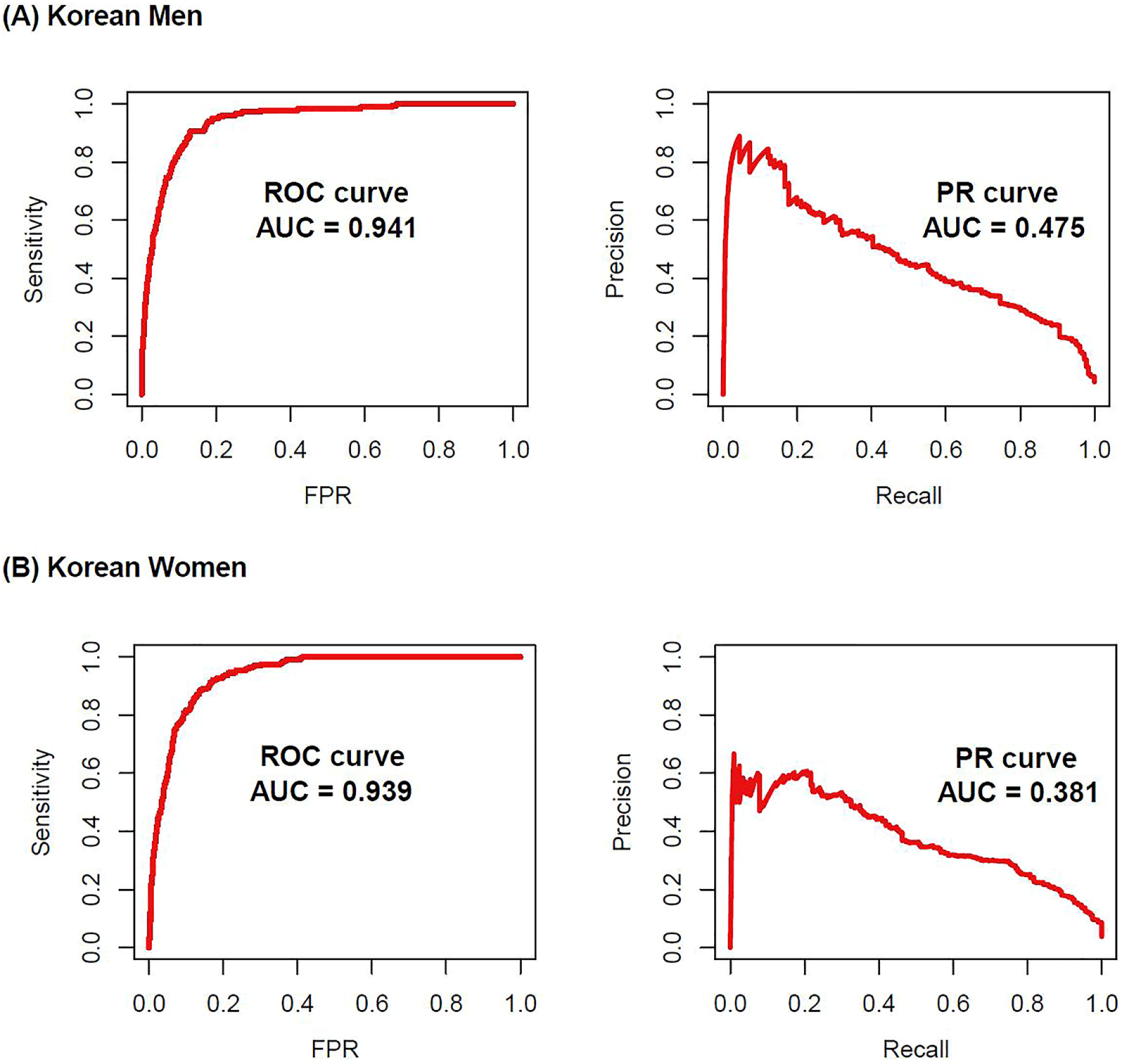
ROC and PR curves for the present gender-specific diabetes prediction model using the KNHANES dataset from 2017 to 2018. (**A**) Men (**B**) women. *KNHANES* Korea National Health and Nutrition Examination Survey, *ROC* receiver operating characteristic, *AUC* area under the curve, *PR* precision recall, *FPR* false positive rate.

We used this gender-specific diabetes classification model to calculate the probabilities of diabetes in subjects from the internal validation dataset. The area under the receiver operating curve (AUROC) was 0.941 and 0.939 in men and women, respectively (Fig. 1). The area of under the precision-recall (PR) curve was 0.475 and 0.381 in men and women, respectively (Fig. 1). Moreover, we evaluated the model performance via calibration, the agreement between observed and predicted probabilities using val.prob function in the rms package. As a result, the classification model for women was a well-calibrated model, besides the model for men was not according to the Spiegelhalter Z-test and its two-tailed *p*-values (S:p for men: 0.008; S:p for women: 0.588, Supplementary Fig. 2).

### External validation of the classification model

**Figure 2:**
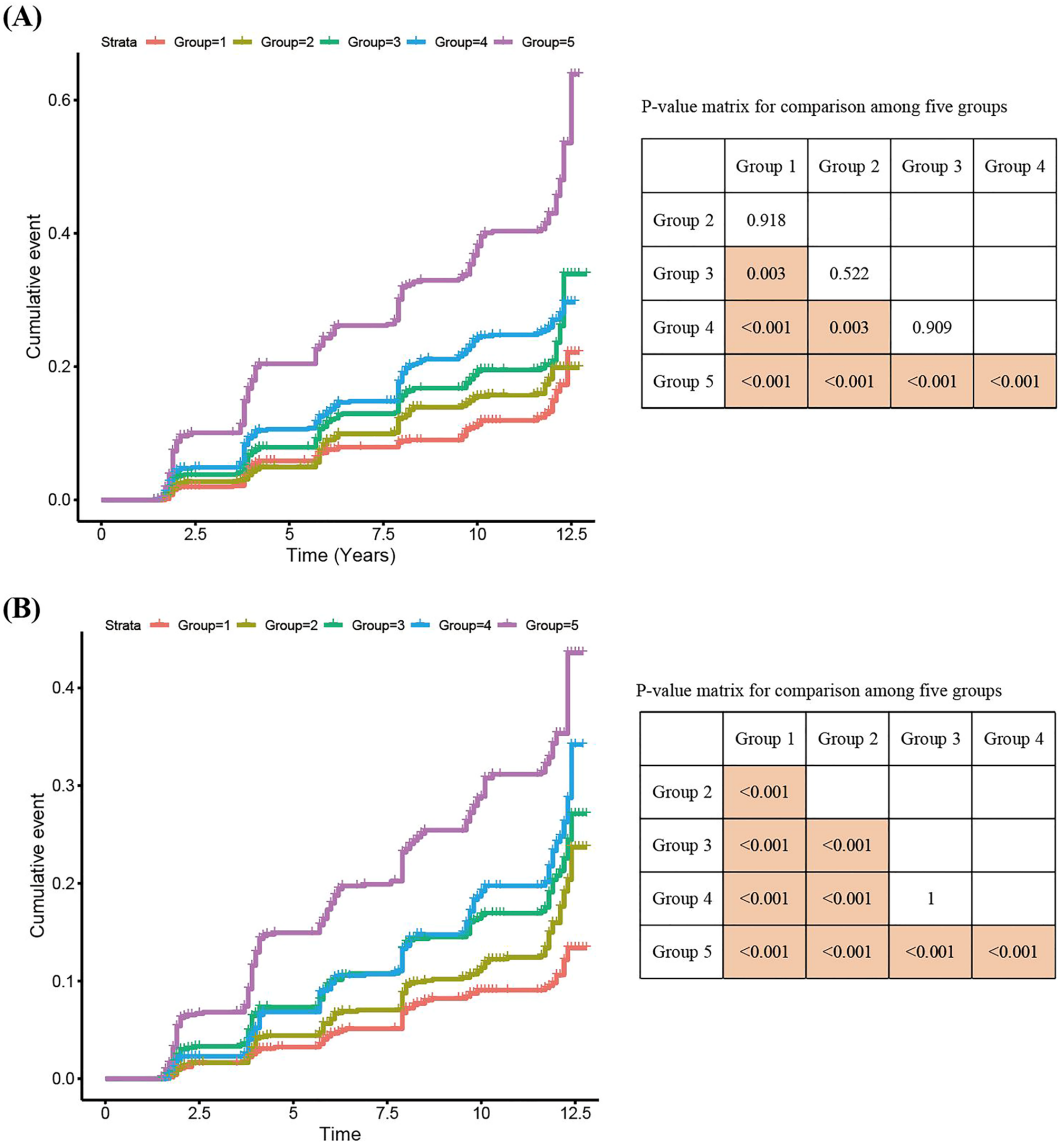
Cumulative incidence difference of new-onset diabetes between five groups, divided according to the expected probabilities of participants in the KoGES study. Group 5 showed highest cumulative incidence in these five groups of both (**A**) men, (**B**) women. *KoGES* Korean Genome and Epidemiology Study.

Table 3 shows baseline characteristics of the KOGES dataset. By using our gender-specific classifying model constructed from the derivation dataset, we calculated the probabilities of the presence of diabetes in subjects from the external validation dataset. These subjects were categorized into five groups according to the probabilities of the subjects in ascending order. Figure 2 shows the cumulative incidence of new-onset diabetes. Most groups had significant differences from other groups. For both men and women, group 5 yielded significantly worse outcomes than those in other groups.

**Table 3:** Baseline characters of external validation set.

	Men			Women		
	Normal	New-onset DM	P-value	Normal	New-onset DM	P-value
N	2662	699		3110	669	
Age, y	50.9 ± 0.17	52 ± 0.32	0.002	51.6 ± 0.16	53.4 ± 0.33	< 0.001
**Income, n**			0.697			0.142
Quartile 1	315 (11.8)	84 (12)		693 (22.3)	175 (26.2)	
Quartile 2	777 (29.2)	208 (29.8)		1025 (33)	216 (32.3)	
Quartile 3	962 (36.1)	262 (37.5)		947 (30.5)	183 (27.4)	
Quartile 4	608 (22.8)	145 (20.7)		445 (14.3)	95 (14.2)	
**Education, n**			0.668			0.001
Elementary	509 (19.1)	124 (17.7)		1284 (41.3)	330 (49.3)	
Middle school	597 (22.4)	163 (23.3)		738 (23.7)	147 (22)	
High school	964 (36.2)	265 (37.9)		876 (28.2)	153 (22.9)	
College	592 (22.2)	147 (21)		212 (6.8)	39 (5.8)	
Smoking, pack years	11.8 ± 0.32	12.8 ± 0.64	0.191	0.3 ± 0.04	0.4 ± 0.12	0.146
Alcohol, g/week	122.5 ± 3.74	136 ± 7.64	0.112	9.3 ± 0.72	9.9 ± 1.51	0.716
Sleep duration, h	6.9 ± 0.02	6.8 ± 0.05	0.189	6.7 ± 0.02	6.7 ± 0.06	0.881
Hypertension, n	258 (9.7)	138 (19.7)	< 0.001	381 (12.3)	153 (22.9)	< 0.001
Dyslipidemia, n	72 (2.7)	30 (4.3)	0.04	44 (1.4)	16 (2.4)	0.096
Cardiovascular disease, n	46 (1.7)	12 (1.7)	0.999	31 (1)	18 (2.7)	0.001
Systolic BP, mmHg	120.7 ± 0.32	125.6 ± 0.65	< 0.001	119.2 ± 0.35	124.7 ± 0.76	< 0.001
Diastolic BP, mmHg	81.4 ± 0.22	84.3 ± 0.42	< 0.001	78.1 ± 0.21	81.3 ± 0.45	< 0.001
**Body mass index, n**			< 0.001			< 0.001
Normal (< 23 kg/m^2^)	984 (37)	186 (26.6)		1012 (32.5)	126 (18.8)	
Pre-obesity (23 ≤ and < 25 kg/m^2^)	732 (27.5)	176 (25.2)		835 (26.8)	159 (23.8)	
Obesity (≥ 25 kg/m^2^)	946 (35.5)	337 (48.2)		1263 (40.6)	384 (57.4)	
Waist circumference, cm	82.6 ± 0.15	85.3 ± 0.29	< 0.001	80.4 ± 0.17	84 ± 0.36	< 0.001
Fasting glucose, mg/dL	83.3 ± 0.16	89.5 ± 0.38	< 0.001	80.4 ± 0.13	84.4 ± 0.35	< 0.001
Glycosuria, n	107 (4)	76 (10.9)	< 0.001	39 (1.3)	25 (3.7)	< 0.001
Total cholesterol, mg/dL	189.9 ± 0.67	195.6 ± 1.31	< 0.001	187.9 ± 0.61	195.1 ± 1.27	< 0.001
Triglyceride, mg/dL	162.9 ± 2.02	199 ± 5.1	< 0.001	133.3 ± 1.24	175 ± 3.61	< 0.001
White blood cell, E3/μL	6.7 ± 0.03	6.9 ± 0.07	0.002	6.2 ± 0.03	6.6 ± 0.07	< 0.001
Hemoglobin, g/dL	14.7 ± 0.02	14.9 ± 0.04	0.001	12.5 ± 0.02	12.7 ± 0.04	< 0.001
Creatinine, mg/dL	0.999 ± 0.0033	1.001 ± 0.0073	0.487	0.7 ± 0.0026	0.7 ± 0.0042	0.971

Continuous and categorical variables are described as mean ± standard error and number (percent), respectively.

P-values are measured using nominal population, not weighted population.

P-values of continuous and categorical variables are measured by Student t-test and Chi-squared test, respectively.

*BP* blood pressure.

## Discussion

Our novel model for the risk classification of diabetes mellitus, based on two large-scale population-based cohort studies, showed high sensitivity and selectivity. Our model yielded AUROCs of 0.941 and 0.939 in men and women, respectively. The Finnish Diabetes Risk Score (FINDRISC) model is a well-known, recommended tool for diabetes mellitus prediction^14^. The AUROC of the FINDRISC model was 0.77 and 0.74 in the Norwegian^15^ and Spanish^16^ populations, respectively. The Framingham Diabetes Risk Scoring Model (FDRSM) by Wilson et al.^17^ yielded an AUROC of 0.85 and 0.78 in middle aged American and Canadian populations, respectively^18^. In the Asian population, Quan Zou et al. predicted new-onset diabetes using the machine learning technique from a Chinese cohort. Their model yielded an AUROC of 0.8084^19^. The diabetes risk score model from the KoGES by Kim et al.^20^ yielded AUROC of 0.71 and 0.76 in men and women, respectively. Note that the predictive performance by our model is for the presence of DM, not the new-onset DM, thereby, somewhat outperforms compared to previous models predicting the new-onset DM. We had performed the literature-review and statistical methods to select more than 15 predictors, which are the potentially appropriate model for DM that has the complex pathophysiology.

With the help of machine learning techniques, we can handle large numbers of participant features that may have positive or negative correlations with the prevalence of diabetes mellitus. To obtain input features for our model, we used data from the KNHANES, a large-scale cross-sectional study that includes approximately 10,000 participants. As a result, we were able to use the 16 and 18 variables in men and women, respectively, during the analysis (Table 3).

Among these variables, glycosuria showed the highest odds ratio (OR) in men (OR 1.35; 95% CI 1.32–1.39). In general, glycosuria has been used as a biomarker for renal complication in diabetes^8,21^, not as a predictor for diabetes. Although glycosuria is a result of hyperglycemia, it also occurs with normal blood glucose levels due to renal injury. Moreover, hyperglycemic patients can also secrete normal range glucose in their urine^22,23^. This implies that we need to identify a new risk factor that, despite being considered negligible, may have a significant impact on predicting diabetes through machine learning techniques. High triglyceride (TG) levels showed the highest OR in women (OR 1.49; 95% CI 1.45–1.54). High TG levels are known to be a result of metabolic dysfunction in patients with diabetes^24^ and a risk factor for diabetes development^25,26^. Recently, a rural Chinese cohort study by Yongcheng et al. reported that hypertriglyceridemia is a risk factor for diabetes^27^. They also suggested that reducing triglycerides can decrease the risk of developing diabetes^27^. This implies that a high TG level is a modifiable risk factor for diabetes and should be managed in people predisposed to diabetes.

Alcohol consumption was related to a decreased risk of diabetes in both men and women (KNHANES dataset). This finding is consistent with previous studies about alcohol consumption. Moreover, heavy and moderate consumption showed deleterious and protective effects on diabetes, respectively^28^. BMI and waist circumference (WC) showed positive relationships in univariate analysis. However, multivariate analysis revealed that BMI had a negative relationship, whereas WC had a positive relationship with diabetes. In light of this, waist circumference, a well-known parameter for central obesity, may be a better parameter for risk assessment of obesity than is BMI, a general obesity indicator. Wang et al. reported similar results regarding risk prediction for diabetes. According to their analysis, abdominal adiposity was superior to abdominal obesity as a predictor for new-onset diabetes^29^. Peter et al. also reported that WC showed higher mortality risk than BMI (WC: HR 1.40 [95% CI 1.14–1.72] and BMI: HR 1.29 [1.04–1.61]) in adults with diabetes^30^.

Risk group classification is one of the most critical uses of machine learning techniques in medical research^31^. Using logistic regression, the combinatory effect of selected risk factors on the disease of interest could be calculated as a probability. Moreover, based on the probability obtained from LR, the participants were classified into five groups. Subsequently, we assessed the risk of each group by analyzing the cumulative incidence of diabetes using cox regression analysis. As expected, and as per our prediction model, participants at high risk showed a high incidence of diabetes (Fig. 2).

Our study had several limitations. First, we could not distinguish type 1 diabetes mellitus from type 2 diabetes mellitus because there were no biomarkers or clinical information for classifying the new-onset diabetes in the KoGES. The risk factors for each type of diabetes are different. Therefore, distinguishing the type of diabetes may be preferable when formulating a prediction model with high accuracy. However, new-onset type 1 diabetes mellitus in a patient over 30 years of age is rare^32^. Hence, this prediction model may be used to classify groups with a high risk for type 2 diabetes mellitus. Second, we could not use menopausal status as a predictive factor in women. The effects of various post-menopausal hormones in women must be considered^33^. Previous cohort studies reported controversial results regarding the role of menopausal status in diabetes development^34,35^. Kim et al. reported that there was no association between natural menopause and the risk for diabetes mellitus^34^. However, early menopause showed significant association with type 2 diabetes mellitus^36^. Unfortunately, KoGES data at baseline did not include the menopausal status of participants. Therefore, we could not use this factor. Third, we used two large cohort composed of Koreans. So, our diabetes index has high generalizability in Koreans, but not high in other populations. However, we had used the nationally representative surveys to establish the DM classification model. Moreover, we validated the model using the KoGES that is also a nation-wide longitudinal study. Due to setting healthy subjects as target population, our model might have the generalizability compared to other models using hospital-based participants.

In conclusion, we developed a diabetes mellitus risk classification model and validated it using Korean datasets. Although the variables used in this model cannot be counted directly, they can be easily collected in real clinical practice. Hence, this new diabetes index can be used to classify individuals at a high risk for diabetes mellitus, who should prevent the disease by managing their risks through lifestyle modification.

## Materials and methods

### Study population

This study used demographic data and biochemical profiles from the 2013–18 KNHANES. The KNHANES is a national surveillance system assessing the health and nutritional status of the Korean population. It is conducted annually by the Korea Centers for Disease Control and Prevention (KCDC). Details of this nationwide survey have been described elsewhere^37^. Subjects aged 40 years and older were included. Subjects with incomplete data regarding demographics and laboratory information were excluded. Furthermore, we excluded subjects with a fasting blood glucose level ≥ 126 mg/dL regardless of a diagnosis of diabetes mellitus. When constructing prediction models, subjects with hyperglycemia may cause bias as this may involve predicting the development of an anticipated pre-existing condition. We determined 2013–16 KNHANES data as the derivation set and 2017–18 KNHANES data as the internal validation set. The target population of KHANES consists of nationally representative non-institutionalized civilians^38^.

The KoGES is an ongoing, prospective, large cohort study conducted by the Korean government. It involves a biannual examination related to life-style surveys, biochemical profiles, and incidences of common chronic diseases of Korean adults since 2001. Details of the KoGES have been described elsewhere^39^. We used the Ansan–Ansung cohort study, a KoGES 10-year data follow-up study, for the external validation set. Subjects who were already diagnosed with diabetes mellitus or exhibited diabetic profiles in lab tests (a fasting glucose level ≥ 126 mg/dL, a 2-h post glucose level ≥ 200 mg/dL in a 75 g oral glucose tolerance test [OGTT], or a glycosylated hemoglobin A1c[HbA1c] level ≥ 6.5%) were excluded at baseline. Finally, 14,977, 9611, and 7140 subjects were used in the derivation, internal validation, and external validation sets for analysis, respectively. The major steps of inclusion/ exclusion processes of this study are described at Supplementary Fig. 1.

### Definition of diabetes

Diabetes was defined according to the American Diabetes Association (ADA) guidelines^40^ as follows: a fasting blood glucose level ≥ 126 mg/dL, a 2-h post glucose level ≥ 200 mg/dL during OGTT, or an HbA1c ≥ 6.5%. Participants who were previously diagnosed as having diabetes or who exhibited diabetic features in their blood samples were categorized as the diabetes group in the KNHANES. In the KoGES, because it is a longitudinal observational study, we included non-diabetic patients in the initial cohort data. Moreover, we detected new-onset diabetes in accordance to the criteria of the ADA during the observation period.

### Variable selection and statistical analysis

To determine predictive risk factors for deriving the risk prediction model, candidate variables were selected based on literature review. Two endocrinologists performed literature review and selected 40 risk factors (Supplementary Table 1). Subsequently, we determined predictive risk factors using backward stepwise logistic regression (LR) method^41^ after applying weight values to all subjects in the KNHANES. Weight values were used for the processes of determining the significant risk factors and deriving the prediction model. These values were determined during data construction and denoted the subjects in the study cohort in which a number of people were represented.

Normal distribution of candidate variables was verified using the Kolmogorovo–Smirnov test. Differences in variables were analyzed based on diabetes status by means of the student’s t-test and Chi-square test for continuous and categorical variables, respectively. Associations between candidate variables were analyzed separately for men and women. The LR model was used to determine the risk factors for the presence of diabetes mellitus, and to formulate the diabetes mellitus prediction model. The AUROC and the Cox regression model were used to measure the performance of the prediction model for the internal validation set and for the external validation set, respectively. Statistical analysis was performed using R language (R packages ver.3.6.1). P-value < 0.05 was considered statistically significant.

### Ethical considerations

The Institutional Review Board of Gwangju Institute of Science and Technology (South Korea) approved the study protocol (IRB No. 20200414-EX-01-02). All research procedures were performed in accordance to the relevant guidelines and regulations. All participants volunteered and provided written informed consent prior to enrolment, and their records were anonymized before being accessed by the authors.

## Supplementary Information

Supplementary Information.
