# Supplementary material for: Development and validation of a new diabetes index for the risk classification of present and new-onset diabetes: multicohort study
Source: Sci Rep. 2021 Aug 3;11:15748. doi: 10.1038/s41598-021-95341-8 (PMC8333254; doi:10.1038/s41598-021-95341-8)
Supplement: Supplementary file 1 — Supplementary Information. [file 41598_2021_95341_MOESM1_ESM.docx]

**Supplementary Table 1.** References in the literature-based search for variables

| **Variables** | **Risk on Diabetes** |
| --- | --- |
| Age | The incidence of diabetes increases with age until about 65 years[1]. |
| Sex | Type 2 diabetes mellitus (T2DM) is more frequently diagnosed at lower age in men[2]. |
| Income | Poverty increase T2DM incidence[3]. |
| Education | Low education level is adversely associated with incidence of diabetes[4]. |
| Smoking | Smoking and nicotine exposure impact body composition, insulin sensitivity, and pancreatic beta cell function[5]. |
| Alcohol | Moderate alcohol consumption is protective for T2DM in men and women[6].  Alcohol drinking frequency is associated with risk of diabetes[7]. |
| Sleep duration | Insufficient sleep duration is the risk factor for T2DM[8]. |
| Hypertension | Hypertension is the independent risk factor for T2DM[9]. |
| Cardiovascular disease | A close link exist between DM and cardiovascular disease[10]. |
| Dyslipidemia | High level of triglyceride and low level of HDL cholesterol are risk factors for T2DM[11]. |
| Menopause | Menopause was not associated with diabetes risk[12]. |
| Stroke | Hyperglycemia confers greater risk of stroke occurrence[13]. |
| Body mass index | Increased BMI was associated with increased prevalence of diabetes, hypertension and dyslipidemia[14]. |
| Waist circumference | Waist circumference and its changes are strongly associated with T2DM[15]. |
| Fasting glucose | An increase in fasting glucose concentration in the nondiabetic range has been shown to be associated with increased risk for T2DM[16]. |
| 1-hr post glucose in oral glucose tolerance test (OGTT), mg/dL | The 1-hr blood glucose level is a stronger predictor of future T2DM than 2-hr blood glucose level[17]. |
| 2-hr post glucose in OGTT, mg/dL | An increase in 2-hr plasma glucose concentration in the nondiabetic range has been shown to be associated with increased risk for T2DM[16]. |
| Family history of diabetes | Family history of diabetes was associated with the incidence of diabetes[18]. |
| Gestational diabetes mellitus (GDM) | The cumulative risk of developing T2DM for the GDM patients was 25.8% at 15 years postdiagnosis[19]. |
| Depression | Depression occurrence is two to three times higher in people with DM[20]. |
| Polycystic Ovary syndrome (PCOS) | The risk of T2DM is markedly elevated in middle-aged women with PCOS[21]. |
| Acanthosis nigricans (AN) | AN is associated with hyperinsulinemia[22]. |
| Glycouria | Urine glucose screening is a feasible method for early detection of asymptomatic T2DM[23]. |
| Physical activity |  |
| Unhealthy diet |  |
| White blood cell | Elevated leukocytes count is associated with chronic complications in T2DM[24]. |
| Hemoglobin, g/dL | Anemia is a major health problem among T2DM[25]. |
| Creatinine, mg/dL | Low serum creatinie is a new risk factor of T2DM[26]. |
| Elevated liver function test | Increased activity of liver enzymes are associated with insulin resistance, metabolic syndrome and T2DM[27]. |

**Supplementary Figure 1.** Flow diagram of the study for DM risk classsification model.


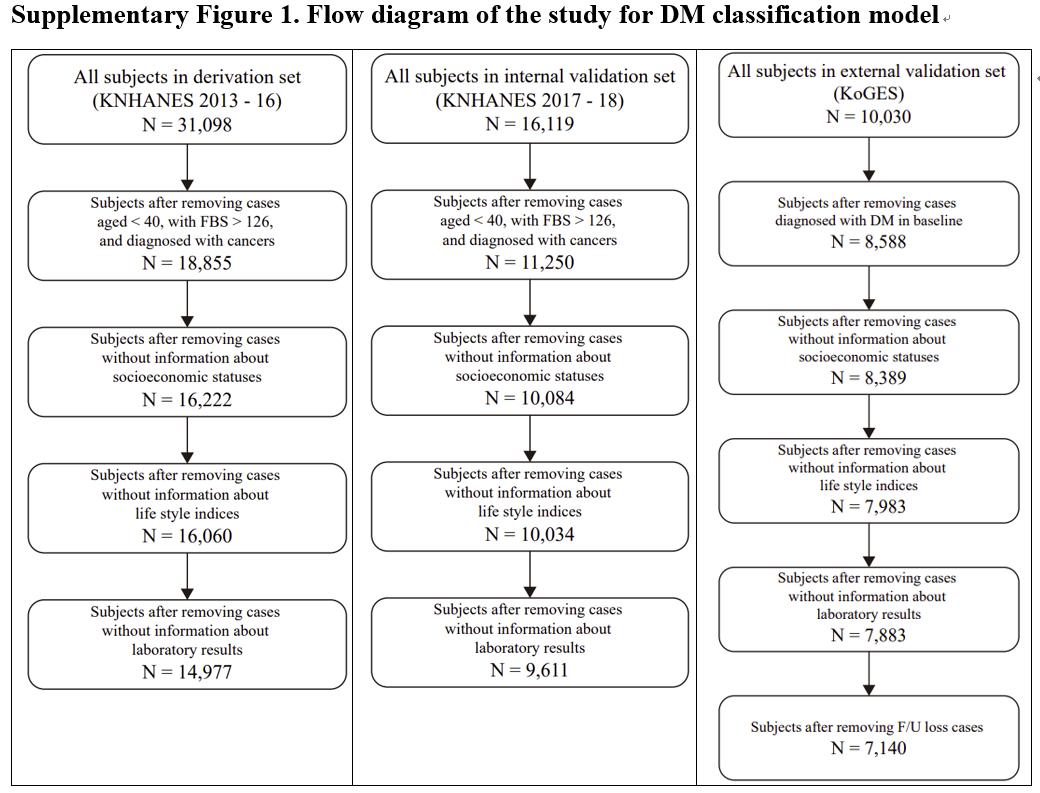


**Supplementary Figure 2.** Calibration plot of the model for test dataset (2017 – 18 KNHANES). Dxy: Somers’ D; D: Discrimination index; U: Unreliability index; Q: quality index; Emax: maximum absolute difference in predicted and loess-calibrated probabilities; S:z and S:p: Spiegelhalter Z-test and its two-tailed p-values.


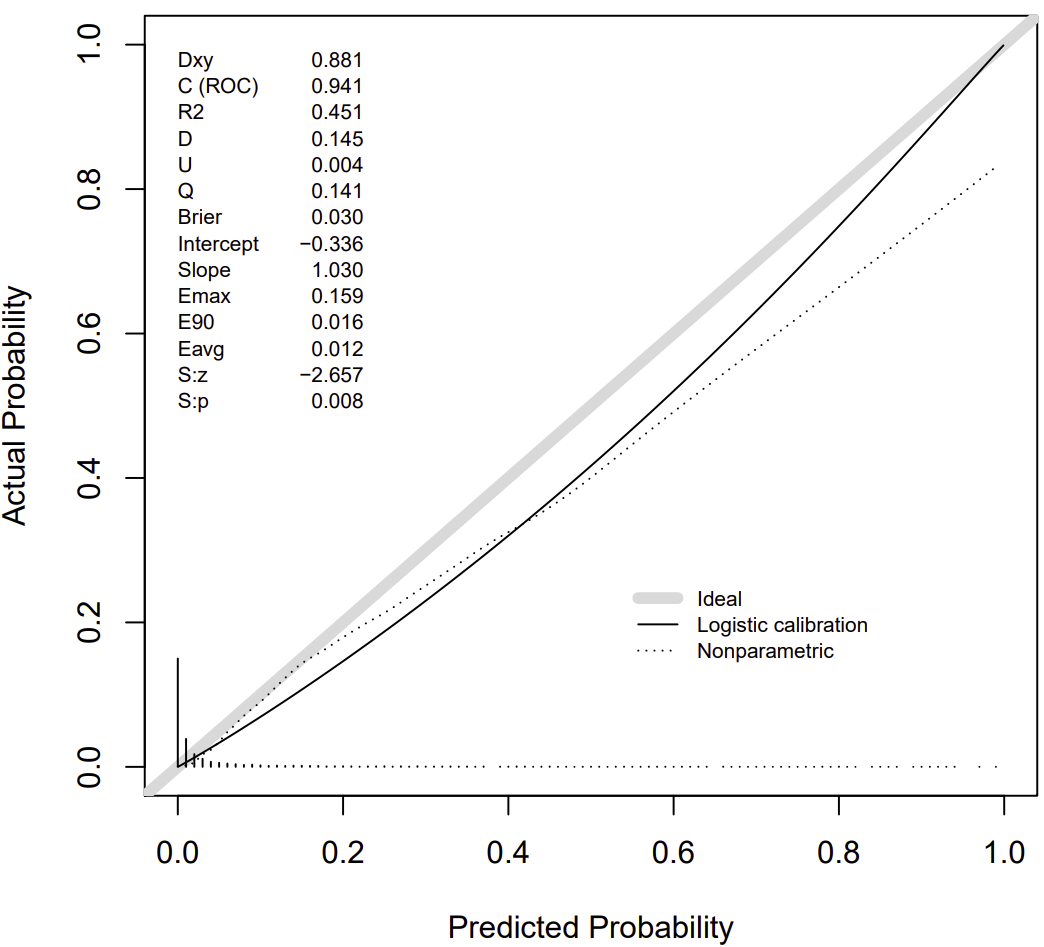


**Men**


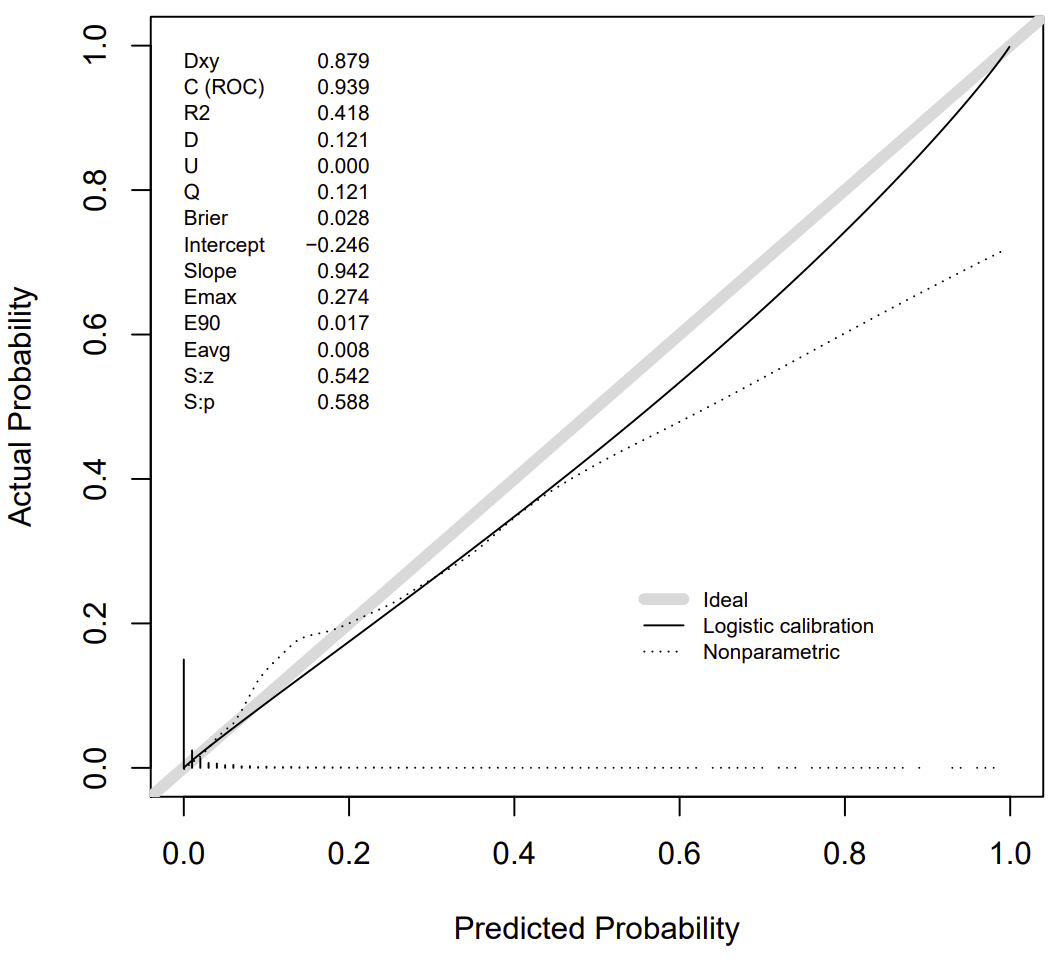


**Women**

**Appendix**

We presented two cases (man and woman) to explain the calculations for the probability of the presence of DM. Two tables included information about the DM risk classification model (Odds ratio and Beta-coefficient).

| Men | Odds ratio | Beta-coefficient | Case 1 |
| --- | --- | --- | --- |
| (Intercept) |  | 8.936 |  |
| Age, y | 1.037 | 0.036 | 55 |
| Income, n | 0.869 | -0.141 | Poor, Quartile 1 (1) |
| Alcohol, g/week | 0.960 | -0.041 | 120 |
| Sleep duration, h | 1.094 | 0.090 | 7 |
| Hypertension† | 1.510 | 0.412 | Presence (1) |
| Dyslipidemia† | 2.647 | 0.973 | Presence (1) |
| Cardiovascular disease† | 1.625 | 0.486 | Absence (0) |
| Systolic BP, mmHg | 0.497 | -0.699 | 130 |
| Diastolic BP, mmHg | 0.512 | -0.670 | 80 |
| Body mass index‡ | 0.770 | -0.262 | 30 (3) |
| Waist circumference, cm | 1.051 | 0.049 | 90 |
| Fasting glucose, mg/dL | 1.090 | 0.086 | 90 |
| Glycosuria† | 6.610 | 1.889 | Absence (0) |
| Total cholesterol, mg/dL | 0.143 | -1.946 | 140 |
| White blood cell, E3/μL | 1.917 | 0.651 | 6.5 |
| Hemoglobin, g/dL | 0.686 | -0.376 | 13 |
| Creatinine, mg/dL | 1.236 | 0.212 | 0.7 |
|  |  |  |  |

$$Linear predictor (LP)=8.936+0.036\times55+\left( -0.141 \right)\times1+\left( -0.041 \right)\times\log_{2} \left( 120 \right)+$$

$$0.090\times7+0.412\times1+0.973\times1+0.486\times0+$$

$$\left( -0.699 \right)\times\log_{2} \left( 130 \right)+ \left( -0.670 \right)\times\log_{2} \left( 80 \right)+\left( -0.262 \right)\times3+$$

$$0.049\times90+0.086 \times90+1.889\times1+\left( -1.946 \right)\times\log_{2} \left( 140 \right)+$$

$$0.651\times\log_{2} \left( 6.5 \right)+\left( -0.376 \right)\times13+0.212\times0.7= -2.12$$

$$Probability= \frac{1}{1+\exp\left( -LP \right)}=\frac{1}{1+\exp\left( -(-2.12) \right)}=0.106$$

| Women | Odds ratio | Beta-coefficient | Case 2 |
| --- | --- | --- | --- |
| (Intercept) |  | 5.833 |  |
| Age, y | 1.053 | 0.052 | 55 |
| Alcohol, g/week | 0.923 | -0.080 | 40 |
| Sleep duration, h | 0.888 | -0.118 | 7 |
| Hypertension† | 1.585 | 0.461 | Presence (1) |
| Dyslipidemia† | 1.621 | 0.483 | Presence (1) |
| Diastolic BP, mmHg | 0.399 | -0.919 | 70 |
| Waist circumference, cm | 1.020 | 0.020 | 68 |
| Fasting glucose, mg/dL | 1.094 | 0.089 | 80 |
| Glycosuria† | 15.074 | 2.713 | Presence (1) |
| Total cholesterol, mg/dL | 0.108 | -2.226 | 110 |
| Triglyceride, mg/dL | 1.306 | 0.267 | 80 |
| White blood cell, E3/μL | 2.698 | 0.992 | 8.5 |
| Hemoglobin, g/dL | 0.730 | -0.315 | 12 |

$$Linear predictor (LP)=5.833+0.052\times55+\left( -0.080 \right)\times\log_{2} \left( 40 \right)+\left( -0.118 \right)\times7+$$

$$0.461\times1+0.483\times1+(-0.919)\times\log_{2} \left( 70 \right)+0.020\times68+$$

$$\left( 0.089 \right)\times80+ 2.713\times1+\left( -2.226 \right)\times\log_{2} \left( 110 \right)+$$

$$0.267\times\log_{2} \left( 80 \right)+0.991 \times\log_{2} \left( 8.5 \right)+\left( -0.315 \right)\times12= -3.245$$

$$Probability= \frac{1}{1+\exp\left( -LP \right)}=\frac{1}{1+\exp\left( -\left( -3.245 \right) \right)}=0.038$$

Case 1 and 2 were estimated as 10.6 and 5.2% of the presence of diabetes, respectively.

**References**

1. Kirkman, M.S., Briscoe, V.J., Clark, N., Florez, H., Haas, L.B., Halter, J.B., Huang, E.S., Korytkowski, M.T., Munshi, M.N., Odegard, P.S.: Diabetes in older adults. Diabetes care **35**(12), 2650-2664 (2012).

2. Kautzky-Willer, A., Harreiter, J., Pacini, G.: Sex and gender differences in risk, pathophysiology and complications of type 2 diabetes mellitus. Endocrine reviews **37**(3), 278-316 (2016).

3. Hsu, C.-C., Lee, C.-H., Wahlqvist, M.L., Huang, H.-L., Chang, H.-Y., Chen, L., Shih, S.-F., Shin, S.-J., Tsai, W.-C., Chen, T.: Poverty increases type 2 diabetes incidence and inequality of care despite universal health coverage. Diabetes care **35**(11), 2286-2292 (2012).

4. Shang, X., Li, J., Tao, Q., Li, J., Li, X., Zhang, L., Liu, X., Wang, Q., Shi, X., Zhao, Y.: Educational level, obesity and incidence of diabetes among Chinese adult men and women aged 18–59 years old: an 11-year follow-up study. PLoS One **8**(6), e66479 (2013).

5. Maddatu, J., Anderson-Baucum, E., Evans-Molina, C.: Smoking and the risk of type 2 diabetes. Translational Research **184**, 101-107 (2017).

6. Baliunas, D.O., Taylor, B.J., Irving, H., Roerecke, M., Patra, J., Mohapatra, S., Rehm, J.: Alcohol as a risk factor for type 2 diabetes: a systematic review and meta-analysis. Diabetes care **32**(11), 2123-2132 (2009).

7. Holst, C., Becker, U., Jørgensen, M.E., Grønbæk, M., Tolstrup, J.S.: Alcohol drinking patterns and risk of diabetes: a cohort study of 70,551 men and women from the general Danish population. Diabetologia **60**(10), 1941-1950 (2017).

8. Grandner, M.A., Seixas, A., Shetty, S., Shenoy, S.: Sleep duration and diabetes risk: population trends and potential mechanisms. Current diabetes reports **16**(11), 106 (2016).

9. Kim, M.-J., Lim, N.-K., Choi, S.-J., Park, H.-Y.: Hypertension is an independent risk factor for type 2 diabetes: the Korean genome and epidemiology study. Hypertension Research **38**(11), 783-789 (2015).

10. Leon, B.M., Maddox, T.M.: Diabetes and cardiovascular disease: Epidemiology, biological mechanisms, treatment recommendations and future research. World journal of diabetes **6**(13), 1246 (2015).

11. Saely, C., Rein, P., Vonbank, A., Zanolin, D., Naerr, G., Leiherer, A., Muendlein, A., Ebner, K.-M., Drexel, H.: High triglycerides, low HDL cholesterol and a low LDL cholesterol per apolipoprotein B ratio predict incident diabetes in patients with established coronary artery disease. In: WIENER KLINISCHE WOCHENSCHRIFT 2015, pp. S153-S153. SPRINGER WIEN SACHSENPLATZ 4-6, PO BOX 89, A-1201 WIEN, AUSTRIA

12. Kim, C., Edelstein, S.L., Crandall, J.P., Dabelea, D., Kitabchi, A.E., Hamman, R.F., Montez, M.G., Perreault, L., Foulkes, M.A., Barrett-Connor, E.: Menopause and risk of diabetes in the Diabetes Prevention Program. Menopause (New York, NY) **18**(8), 857 (2011).

13. Chen, R., Ovbiagele, B., Feng, W.: Diabetes and stroke: epidemiology, pathophysiology, pharmaceuticals and outcomes. The American journal of the medical sciences **351**(4), 380-386 (2016).

14. Bays, H.E., Chapman, R., Grandy, S., Group, S.I.: The relationship of body mass index to diabetes mellitus, hypertension and dyslipidaemia: comparison of data from two national surveys. International journal of clinical practice **61**(5), 737-747 (2007).

15. Fan, Y., Wang, R., Ding, L., Meng, Z., Zhang, Q., Shen, Y., Hu, G., Liu, M.: Waist Circumference and its Changes Are More Strongly Associated with the Risk of Type 2 Diabetes than Body Mass Index and Changes in Body Weight in Chinese Adults. The Journal of Nutrition **150**(5), 1259-1265 (2020).

16. Abdul-Ghani, M.A., DeFronzo, R.A.: Plasma glucose concentration and prediction of future risk of type 2 diabetes. Diabetes Care **32**(suppl 2), S194-S198 (2009).

17. Pareek, M., Bhatt, D.L., Nielsen, M.L., Jagannathan, R., Eriksson, K.-F., Nilsson, P.M., Bergman, M., Olsen, M.H.: Enhanced predictive capability of a 1-hour oral glucose tolerance test: a prospective population-based cohort study. Diabetes care **41**(1), 171-177 (2018).

18. Sakurai, M., Nakamura, K., Miura, K., Takamura, T., Yoshita, K., Sasaki, S., Nagasawa, S.y., Morikawa, Y., Ishizaki, M., Kido, T.: Family history of diabetes, lifestyle factors, and the 7‐year incident risk of type 2 diabetes mellitus in middle‐aged Japanese men and women. Journal of diabetes investigation **4**(3), 261-268 (2013).

19. Lee, A.J., Hiscock, R.J., Wein, P., Walker, S.P., Permezel, M.: Gestational diabetes mellitus: clinical predictors and long-term risk of developing type 2 diabetes: a retrospective cohort study using survival analysis. Diabetes care **30**(4), 878-883 (2007).

20. Bădescu, S., Tătaru, C., Kobylinska, L., Georgescu, E., Zahiu, D., Zăgrean, A., Zăgrean, L.: The association between diabetes mellitus and depression. Journal of medicine and life **9**(2), 120 (2016).

21. Gambineri, A., Patton, L., Altieri, P., Pagotto, U., Pizzi, C., Manzoli, L., Pasquali, R.: Polycystic ovary syndrome is a risk factor for type 2 diabetes: results from a long-term prospective study. Diabetes **61**(9), 2369-2374 (2012).

22. Stoddart, M.L., Blevins, K.S., Lee, E.T., Wang, W., Blackett, P.R.: Association of acanthosis nigricans with hyperinsulinemia compared with other selected risk factors for type 2 diabetes in Cherokee Indians: the Cherokee Diabetes Study. Diabetes care **25**(6), 1009-1014 (2002).

23. Kim, M.S., Lee, D.-Y.: Urinary glucose screening for early detection of asymptomatic type 2 diabetes in Jeonbuk province Korean schoolchildren. Journal of Korean Medical Science **32**(6), 985-991 (2017).

24. Naredi, M.: Study of relationship between WBC count and diabetic complications. International Journal of Advances in Medicine **4**(4), 1128 (2017).

25. Bekele, A., Teji Roba, K., Egata, G., Gebremichael, B.: Anemia and associated factors among type-2 diabetes mellitus patients attending public hospitals in Harari Region, Eastern Ethiopia. PloS one **14**(12), e0225725 (2019).

26. Harita, N., Hayashi, T., Sato, K.K., Nakamura, Y., Yoneda, T., Endo, G., Kambe, H.: Lower serum creatinine is a new risk factor of type 2 diabetes: the Kansai healthcare study. Diabetes care **32**(3), 424-426 (2009).

27. Cho, N.H., Jang, H.C., Choi, S.H., Kim, H.R., Lee, H.K., Chan, J.C., Lim, S.: Abnormal liver function test predicts type 2 diabetes: a community-based prospective study. Diabetes care **30**(10), 2566-2568 (2007).
